# New Mechanisms of Action of Natural Antioxidants in Health and Disease II

**DOI:** 10.3390/antiox10081200

**Published:** 2021-07-27

**Authors:** Silvana Hrelia, Cristina Angeloni

**Affiliations:** 1 Department for Life Quality Studies, Alma Mater Studiorum, University of Bologna, Corso d’Augusto 237, 47921 Rimini, Italy; silvana.hrelia@unibo.it; 2 School of Pharmacy, University of Camerino, Via Gentile III da Varano, 62032 Camerino, Italy

Epidemiological studies have demonstrated an association between natural antioxidants and human health [1], suggesting that they are mainly effective in counteracting noncommunicable diseases such as neurodegenerative and cardiovascular diseases, diabetes, and cancer [2,3,4,5]. The mechanisms by which natural antioxidants exert their health protective activity have been extensively revised in recent years. In particular, the classical direct scavenging activity in which natural antioxidants react in one-electron reactions with free radicals has been set aside because kinetic constraints indicate that in vivo scavenging of radicals is ineffective in antioxidant defense [6]. One of the most studied mechanisms by which natural antioxidants exert their protective effect is the activation of the Nrf2 (NF-E2-related factor 2) signaling pathway, which up-regulates the endogenous antioxidants defense system boosting the expression of antioxidants and detoxifying enzymes (reviewed in [7]). In addition, natural antioxidants exert actions that go beyond their ability to counteract oxidative stress. For many natural antioxidants, the antioxidant effects may be less important for health than other effects including the modulation of different targets such as intracellular signaling cascades [8,9,10], epigenetic mechanisms [11,12,13] and the gut microbiota [11,14,15]. 

Moreover, an important issue that must be taken into consideration studying the protective activity of natural antioxidants is their in vivo bioavailability. Natural antioxidants such as polyphenols demonstrate low bioavailability because of different factors: interaction with the food matrix, intestine uptake, microbiota interaction, and endogenous transformation by phase I and phase II metabolism [16]. On the other hand, many of the activities exerted by natural antioxidants may be mediated by their metabolites, which are produced in vivo [17,18].

This Special Issue, concerned with new mechanisms of action of natural antioxidants in health and disease, contains eighteen contributions: ten research articles and eight reviews, addressing the most recent advances on this topic.

The intake of natural antioxidants can have a profound impact on DNA damage induced by oxidative stress. To study this aspect, Del Bo et al. [19] enrolled 49 older subjects with intestinal permeability to investigate the association among the level of DNA damage (evaluated as DNA strand-breaks, endogenous and oxidatively induced DNA damage) with clinical, metabolic and dietary markers. Interestingly, men showed a higher level of DNA damage compared to women. DNA damage was also positively associated with clinical/metabolic markers (e.g., uric acid, lipid profile) and inversely to dietary markers (e.g., vitamin C, E, B6, folates) and, also for these parameters, there were differences based on sex. Taking into consideration the importance of DNA stability during aging, these aspects should be further investigated in a larger group of older adults to confirm the associations found and to promote preventive strategies.

Martinelli et al. [20] studied the effects of the treatment with (+/−)-alpha-lipoic acid (ALA) and its enantiomers on renal and heart parenchyma in spontaneously hypertensive rats (SHR). ALA is a natural antioxidant present in mitochondria where it acts as coenzyme for pyruvate dehydrogenase and α-ketoglutarate dehydrogenase. The antioxidant properties of ALA are associated with its redox active disulfide group. The authors found greater effectiveness of (+)-ALA compared to (+/−)-ALA in counteracting oxidative stress as demonstrated by the reduction of oxidized proteins levels, and by 8-oxo-dG expression, as well as cardiac and renal damages induced by hypertension. They suggested that the higher effectiveness of (+)-ALA compared to the racemic form could be related to a higher bioavailability that enhanced the antioxidant activity in SHR animals. 

Oxidative stress is a common hallmark of several neurodegenerative diseases and, for this reason, many studies focused on the possibility to counteract neurodegeneration by antioxidants. Extra-virgin olive oil (EVOO) is a rich source of antioxidant phenols with potential activity against neurodegeneration [21]. The study of Barbalace et al. [22] investigated the antioxidant and neuroprotective activity of two different EVOO extracts obtained from the same cultivar trees (Quercetano) but grown in different areas (plain and hill) of the Tuscany region (Italy). Of note, the different areas of the orchards influenced the phenol pattern of the extracts obtained from the oils. Plain extract presented a higher content of phenyl ethyl alcohols, cinnammic acids, oleacein, oleocanthal and flavones; meanwhile, hill extract was richer in lignans. Of course, these differences influenced the bioactivity of the extracts. Hill extract was more effective than plain extract in counteracting oxidative stress in differentiated neuron-like SH-SY5Y cells and this protection was mediated by the up-regulation of the antioxidant enzymes heme oxygenase 1, NADPH quinone oxidoreductase 1, thioredoxin reductase 1 and glutathione reductase. In addition to the antioxidant activity, proteomic analysis revealed that hill extract plays a role in the regulation of proteins involved in neuronal plasticity and activation of neurotrophic factors such as brain derived neurotrophic factor (BDNF). 

Another class of promising neuroprotecting phenols are coumarins, which can be found in medicinal plants such as *Cichorium intybus*, *Artemesia capillaris*, *Ceratostigma willmottianum* and *Citrus limonia*. Using the same cellular model of the previous study, Pruccoli et al. [23] studied the neuroprotective activity of different coumarins (esculetin, scopoletin, fraxetin and daphnetin) and demonstrated that esculetin (ESC) was the most effective in preventing and counteracting reactive oxygen species (ROS) production in neuronal SH-SY5Y cells, suggesting its ability to act as direct and indirect antioxidant through the activation of Nrf2. Moreover, ESC protected SH-SY5Y cells from oligomers of A1–42 peptides induced damage and this protection seems to be mediated by Erk1/2 and Akt signaling pathways confirming, once more, that natural antioxidants possess other activities in addition to their ability to neutralize ROS.

Tonolo et al. [24] focused on the antioxidant properties of bioactive peptides present in fermented soy products subjected to an in vitro digestion in Caco-2 cell line. In vitro digestion mimics the conditions that occur physiologically in the human gastro-intestinal tract. The results demonstrated that several peptides were able to activate the Keap1/Nrf2 pathway with the consequent overexpression of antioxidant and phase II enzymes such as superoxide dismutase 1, thioredoxin reductase 1, glutathione reductase, and NADPH Quinone oxidoreductase 1 (NQO1). These data suggest that, once they are released from the native protein, some bioactive peptides display antioxidant properties protecting the body against oxidative stress.

Sclareol is one of the compounds found in Salvia sclarea, is a bicyclic diterpene alcohol and its antioxidant and anti-inflammatory activities have been already demonstrated. On these bases, Wong et al. [25] investigated the anti-dysmenorrhea activities of sclareol in ex vivo and in vivo dysmenorrhea models, focusing also on its mechanism of action. They found evidence that sclareol reduced prostaglandin (PG) F2α-, oxytocin-, acetylcholine-, carbachol-, KCl-, and Bay K 8644-induced uterine contraction and showed an analgesic effect. Sclareol influenced the Ca^2+^ level and modulated oxytocin receptor (OTR), myosin light chain kinase (MLCK), extracellular signal-regulated kinase, p-p38, cyclooxygenase-2 (COX-2), and phospho-myosin light chain 20 (p-MLC20) protein expression. These data suggest that the natural antioxidant sclareol could be also an effective supplement for dysmenorrhea.

*Tartary buckwheat* (TB, *Fagopyrum esculentum*) flour is used to produce noodles, gels, and breads and it is rich in flavonoids, especially rutin (quercetin-3-0-rutinoside). TB hull and bran are richer in rutin and quercetin compared to TB flour and, for this reason, Jin et al. [26] enhanced rutin content in TB flour extract (TBFE) through hydrothermal treatments and investigated the pharmacokinetic profiles of native TBFE, three differently hydrothermal treated (autoclaving, boiling, or steaming) TBFEs, and standard rutin after a single-dose oral administration in rats. As expected, rutin-enriched TBFEs had a better oral absorption and were retained longer in the bloodstream than native TBFEs or standard rutin. Moreover, they demonstrated a protective effect of TBFEs against ethanol-induced liver injury in rats that received the extracts for 28 days prior to ethanol administration. Rutin-enriched TBFEs triggered a higher increase in antioxidant enzymes and intracellular antioxidant levels protecting the liver against injury caused by repetitive ethanol administration, as confirmed by analyzing relative liver weight, liver injury markers, lipid peroxidation, and calcium permeability. 

Honokiol is a lignan widely distributed in the barks, seed cones, and leaves of the *Magnolia tree* with antioxidant properties. Although Honokiol might be effective in counteracting oxidative stress, it possesses a low bioavailability. To overcome this limitation, Wang et al. [27] encapsulated Honokiol into nanosized liposome and evaluated its efficacy against cisplatin-induced testicular injury. They demonstrated both in vitro and in vivo that nanosome-encapsulated honokiol attenuated cisplatin-induced DNA oxidative stress by suppressing intracellular ROS production and elevating gene expressions of mitochondrial antioxidation enzymes. Nanosome honokiol also mitigated endoplasmic reticulum stress through down regulation of Bip-ATF4-CHOP signaling pathway. Additionally, this natural polyphenol compound diminished cisplatin-induced DNA breaks and cellular apoptosis.

*Lotus* (Nelumbo nucifera Gaertn.) is a perennial flowering plant found in nature in bodies of fresh water. Almost all parts of lotus are edible, meanwhile lotus seedpod is one of its by-products that has been reported to be rich in phenolic compounds with antioxidant activity. Lee et al. [28] demonstrated the pancreatic beta-cell protective effects of a lotus seedpod aqueous extract (LSE) against oxidative injury. By HPLC/ESI-MS-MS method, they showed that the main flavonoid present in LSE is quercetin-3-glucuronide (Q3G). Using rat pancreatic beta-cells, they observed that LSE counteracts oxidative stress induced by H_2_O_2_ increasing cell viability and reducing apoptosis via phospho-Bcl-2-associated death promoter (p-Bad)/B-cell lymphoma 2 (Bcl-2) and reducing the impairment of insulin secretion. The beta-cell protective effect of LSE was carried out by activating autophagy via class III phosphatidylinositol-3 kinase (PI3K)/LC3-II signal pathway. Interestingly, comparable results were obtained using pure Q3G suggesting that LSE protective effects could be mainly mediated by this compound. They also conducted in vivo experiments using high-fat diet (HFD) combined with the streptozotocin (STZ)-induced diabetic mice model demonstrating that the administration of LSE improved the levels of serum biochemical parameters and diabetes mellitus (DM) symptoms, as well as strongly reduced the expressions of apoptosis protecting pancreatic tissue of HFD/STZ-induced DM mice.

Coenzyme Q10 (CoQ10) is an endogenous lipophilic molecule formed by a benzoquinone head conjugated to a side chain composed by 10 isoprene units. Ubiquinone plays crucial physiological functions in the body participating in the electron transport chain in the mitochondria and protecting cells against oxidative stress. Many studies have demonstrated that the supplementation of CoQ10 can counteract many cutaneous disorders even if its low bioavailability can impair its efficacy. In this scenario, Sguizzato et al. [29] designed and characterized phosphatidylcholine based ethosomes (ETHO) containing CoQ10 and investigated the behavior of CoQ10 loaded in ETHO in primary human skin fibroblasts and in reconstituted human epidermis (RHE). Notably, due to their peculiar supramolecular organization, ETHO were able to increase the entrapment capacity of CoQ10 and to better control its stability with respect to other nanosystems previously investigated in other studies. Transmission Electron Microscopy analyses confirmed the ETHO fibroblasts uptake, as well as their passage through the more complex model RHE. Moreover, the pretreatment with CoQ10 loaded in ETHO exerted a consistent protective effect against oxidative stress, in both ex vivo models.

The reviews published in this Special Issue address different aspects related to natural antioxidants focusing on their bioavailability, supplementation to counteract specific diseases and their use in particular animal models.

Regarding bioavailability, Cosme et al. [30] discussed the crucial role of the bioavailability of plant phenolic compounds to accomplish their health effect and highlighted the main factors that influence bioavailability such as absorption, metabolism and excretion. In addition, they gave an updated overview of the potential positive health effects of phenolic compounds mainly related to their direct and indirect antioxidant activity and analyzed the most recent systems to improve phenols bioavailability in humans.

Maldonado et al. [31] reviewed the use of natural antioxidants as co-adjuvant therapy to treat Chagas disease, one of the most prevalent tropical diseases in Latin America that causes the death of several thousand people each year. Natural antioxidants could represent an important help to manage this disease given that no effective treatment has been found. Nevertheless, the authors underlined that antioxidant worked successfully only in experimental models of Chagas disease and their efficacy in patients has not been established yet. 

Ungurianu et al. [32] focused on the antioxidant vitamin E, analyzing its role in the modulation of signaling pathways linked to inflammation and malignancy, highlighting how this group of molecules possesses promising potential for the prevention and treatment of diseases with an inflammatory, redox, or malignant component.

Hsu et al. [33] addressed a very important aspect of antioxidant supplementation: timing. In particular, they concentrated on the prenatal period when the exposure to excessive ROS generated by adverse in utero conditions can cause developmental programming of hypertension. In this scenario, natural antioxidants can play a critical role reversing programming processes and preventing hypertension of developmental origin. Therefore, the use of supplements containing natural antioxidants should be suggested to pregnant mothers in order to reduce their children’s risk for hypertension later in life. Budani and Tiboni [34] also focused on the role of natural antioxidants in the prenatal period and, in particular, on the potential benefit of natural antioxidants in the optimization of infertility treatments. The authors, taking into consideration both experimental studies on oocytes/embryos and clinical trials on antioxidants supplementation, concluded that antioxidant supplementation could have a positive impact on in vitro fertilization outcomes in terms of quality and cryotolerance of in vitro produced embryos, together with positive effects on in vitro maturation oocytes and on early embryonic development.

Garcia-Medina et al. [35] analyzed the role of natural antioxidant in counteracting glaucoma, a neurodegenerative disease characterized by the progressive degeneration of retinal ganglion cells in which oxidative stress plays a critical role. They underlined that recently different studies investigated the effect of the combination of several antioxidants rather than supplementing a single compound. This approach presents pros and cons as it permits to simultaneously modulate multiple targets even if it is difficult to find out the exact effect of each antioxidant when combined. Nevertheless, supplementation with antioxidants may be a promising therapy in glaucoma.

Another paper from the same research group reviewed in vitro experiments, animal studies and clinical trials dealing with the effect of the antioxidants on diabetic retinopathy, another disease where oxidative stress covers a crucial role [36]. Current therapies for diabetic retinopathy do not completely stop the evolution of this disorder underling the importance of alternative strategies like antioxidant supplementation. 

Carillo et al. [37] summarized the current knowledge on L-carnitine in *Drosophila melanogaster*, also known as fruit fly, and discussed the role of the L-carnitine pathway in fly models of neurodegeneration. L-Carnitine is an amino acid derivative that can also act as antioxidant reducing oxidative damage. Drosophila can biosynthesize L-carnitine, and its carnitine transport system is similar to the human transport system; moreover, aberrant regulation of genes involved in carnitine biosynthesis and mitochondrial carnitine transport in Drosophila models has been linked to neurodegeneration. For these reasons, Drosophila models could be useful to study the links between L-carnitine and the development of neurodegenerative disorders.

In conclusion, this Special Issue adds new information to the field of natural antioxidants demonstrating their promising role in supporting health by mechanisms which go well beyond their well-known antioxidant effect.
